# The Institutional Worker

**Published:** 1912-05-25

**Authors:** 


THE
### INSTITUTIONAL WORKER
**Being a Special Supplement to "The Hospital"**

### Editor's Notices.

**Contributions:**

Contributions should be written, or preferably typed, on one side of the paper only, and all articles sent in are accepted upon the distinct understanding that they are forwarded to THE HOSPITAL only.

The Editor cannot undertake to return MSS. not used, but when a stamped directed envelope is enclosed the MSS. may be returned if a special request is made.

Accepted articles and paragraphs of news will be paid for after publication at the scale rate.

**Address:**

To prevent delay all contributions and letters on editorial business must be addressed exclusively to the Editor, "The Hospital," 29 Southampton Street, Strand, London, W.C. It is important that this regulation shall be strictly observed.

**Correspondence:**

Correspondence on all subjects is invited. The name and address of correspondents must be given as a guarantee of good faith, but not necessarily for publication.

**Special Articles:**

Special articles are invited, and questions, inquiries, and paragraphs upon all matters relating to the work, administration, and management of general, special, mental, fever, cottage and convalescent hospitals, sanatoria, homes, institutions, societies and organisations for the treatment or care of the sick, injured and dependants of all classes. Every matter affecting the interests and welfare of the staff of all grades working in these institutions will receive special consideration.

**Administrative Medicine, Research and Items of News:**

Special terms will be paid for approved articles on subjects in this wide field by experts who have made some section of it their special study and interest. We wish to give prominence to every movement tending to advance accuracy of diagnosis, efficiency in treatment, and the development of modern methods to eradicate disease and promote the welfare of the suffering.

**A Bureau of Information:**

We invite inquiries and applications for information and help in providing for that numerous class of sufferers who require special care or house-room which they cannot obtain in their own homes or secure for themselves. **We cannot, however, prescribe, or recommend practitioners.**

**Books for Review:**

Publishers are particularly requested to send advance proofs of any new books of importance whenever possible, as the Editor has made arrangements to publish immediate reviews on a new plan.

**Photographs, Plans, Blocks, and Illustrations:**

It is requested that wherever possible MSS. may be accompanied by illustrations in any of the above forms. The name of the sender and of the article to which it belongs should be written on the back of each photograph, drawing, or block for purposes of identification.

**Local Papers:**

Newspapers containing reports or news paragraphs **should be marked** and addressed to the Sub-Editor.

**The Coupon System:**

A coupon will be found at the bottom of the third inside page of the cover each week. This coupon must be attached to every question or inquiry to which an answer is desired in THE HOSPITAL.

### The Workers' Journal.

THERE is no Journal other than THE HOSPITAL that so comprehensively covers the whole Institutional World. It is not only essential to those responsible for the Construction, Administration, and Maintenance of Institutions generally, but also to the individual worker in every department of the Medical, Health, and Sanitary Services. Useful and authoritative articles on Institution, Poor-Law, and Municipal affairs, as well as the all-important question of National Insurance are contained within its columns. To keep abreast, therefore, with the progress in all branches of Institutional Work, it is necessary to secure a copy of THE HOSPITAL every week; to miss even one Number is an irreparable loss, which no keen worker can afford. In these circumstances, it is advisable to place a permanent order with your Newsagents, so as to ensure that you obtain a copy of THE HOSPITAL every Thursday, or a subscription may be forwarded to the Publishers at the undermentioned rates.

**Subscriptions,** which may commence at any time, are payable in advance. Cheques and Post-Office Orders should be crossed London County and Westminster Bank, Covent Garden Branch, and made payable to the Manager of THE HOSPITAL.

RATES OF SUBSCRIPTION (payable in advance).

| | |
|---|---|
| **UNITED KINGDOM.** | |
| Three Months (including Postage) ... ... ... ... ... | 2s. 0d. |
| Six Months do. ... ... ... ... ... | 4s. 0d. |
| Twelve Months do. ... ... ... ... ... | 6s. 6d. |
| **FOREIGN AND COLONIAL.** | |
| Three Months (including Postage) ... ... ... ... ... | 2s. 6d. |
| Six Months do. ... ... ... ... ... | 5s. 0d. |
| Twelve Months do. ... ... ... ... ... | 8s. 8d. |

*SUBSCRIPTION FORM.*

*Please send me* THE HOSPITAL *for*......................*months, commencing with your issue of*................................, *for which please find enclosed*..............................

*Name*............................................

*Address* ..............................................

..............................................

*Date*.................................

*To the Manager,*
*"The Hospital" Building,*
*28 & 29 Southampton Street,*
*Strand, London, W.C.*

### Manager's Notices.

**All letters on business matters must be addressed to The Manager, "The Hospital," The Hospital Building, 28 and 29 Southampton Street, London, W.C.**

**Advertisements:**

To ensure insertion, all advertisements for THE HOSPITAL must reach the Manager not later than Wednesday morning in each week. For scale of charges see pages vii and 2.

**Special Rates** will be quoted for those Institutions that agree to send all their vacancy, contract, and public notices to THE HOSPITAL each year, so as to make it a file of such announcements for ready reference.

**Remittances:**

**It is especially requested that remittances be made by Cheque or Postal Order, and in halfpenny stamps only when the amount is under sixpence.**

### OFFICIAL APPOINTMENTS VACANT.

### Poor-Law Vacancies.

Assistant Charge Mistress (£40), West London School District. Mr. F. G. Beeching, Clerk to the Managers, Ashford, Middlesex. May 30.

Assistant Master (£70), West Ham Union. Mr. T. Smith, C. to G., Leytonstone, N.E. June 5.

Assistant Matron (£25), Christchurch Union. Mr. A. Druitt, C. to G., Christchurch. May 30.

Assistant Nurse (£20), Dover Union. Mr. E. Carder, C. to G., Dover. May 28.

Assistant Nurse (£25), Bishop's Stortford Union. A. G. Gwynn, C. to G., Bishop's Stortford. May 27.

Assistant Nurse (£20), Kingsclere Union. Mr. J. Barnes, C. to G., Newbury. May 27.

Assistant Nurses (£16), Thorne Union. Mr. G. H. Newborn, C. to G., Thorne, Doncaster. May 29.

Assistant Nurse (£25), Exeter Guardians. Mr. A. Snell, C. to G., Exeter. May 29.

Assistant Nurse (£25), Oxford Guardians. The Master, Workhouse, Cowley Road, Oxford. May 28.

Assistant Nurse (£20), Bideford Union. M. J. Durant, C. to G., Bideford. May 28.

Assistant Nurses (£25), Wisbech Union. T. H. Stockdale, C. to G., 2 Ely Place, Wisbech. June 1.

Assistant Relieving Officers (£125), Birmingham Union. Mr. R. J. Curtis, C. to G., Birmingham. May 28.

Assistant Tailor, etc. (£35), Portsmouth Guardians. Mr. E. H. Mitchell, C. to G., Portsmouth. May 27.

Barber, etc. (£30), Ormskirk Union. Mr. A. Dickinson, Ormskirk. June 5.

Charge Nurse (£27 10s.), Shepton Mallet Union. Mr. A. E. Nalder, C. to G., Shepton Mallet. May 30.

Charge Nurse (£27 10s.), Whitby Union. Mr. W. S. Gray, C. to G., Whitby. May 31.

Charge Nurse (£35), Hull Guardians. R. H. Winter, C. to G., St. Mary's Chambers, Lowgate, Hull. June 4.

Charge Nurse (£30), Keighley Union. S. Green, C. to G., Keighley. May 27.

Charge Nurse (£30), Steyning Union. A. Flower, C. to G., Union Offices, Shoreham-by-Sea. May 27.

Charge Nurse (£30), Ecclesall Bierlow Union. J. E. Moulding, C. to G., Union Office, The Edge, Sheffield. May 29.

Charge Nurse (32), Dorking Union. Mr. W. James, C. to G., Dorking. May 30.

Charge Nurse (£28), Prescot Union. A. F. Mann, C. to G., Union Offices, Whiston, Prescot. May 31.

Dispensary Porter (9s. weekly), Hammersmith Guardians. The C. to G., 206 Goldhawk Road, Shepherd's Bush, W. May 27.

Female Attendant (£26), Bucklow Union. The C. to G., Knutsford. May 27.

Female Attendants (£30), Reading Guardians. Mr. W. H. Oliver, C. to G., Reading. June 12.

Foster-Mother (£25), Pocklington Union. Mr. T. Robson, C. to G., Pocklington. May 31.

Foster-Mother (£25), Maldon Union. Mr. A. W. Freeman, C. to G., Maldon, Essex. May 28.

Foster-Mother (£25), York Union. Mr. G. Sykes, C. to G., York. May 27.

General Assistant (£25), Bucklow Union. The C. to G., Knutsford. May 27.

Head Male Imbecile Attendant (32s. 6d. weekly, non-resident), Wigan Union. Mr. H. G. Ackerley, C. to G., Wigan. May 30.

Infirm Women's Attendant (£25), North Bierley Union. Mr. W. G. Cooper, C. to G., 4 Town Hall Street, Bradford. May 30.

Lady Clerk (27s. 6d. weekly, non-resident), West London School District. Mr. F. G. Beeching, Clerk to the Managers, Ashford, Middlesex. June 3.

Laundress (£30), Newbury Union. Mr. S. V. Pinniger, C. to G., Newbury. May 29.

Master and Matron (£60 and £35), Blything Union. Mr. H. Mullens, C. to G., Bulcamp, Halesworth, Suffolk. May 27.

Master's Assistant (£26), Bishop's Stortford Union. Mr. A. G. Gwynn, C. to G., Bishop's Stortford. May 27.

Male Cook (£35), Blackburn Union. Mr. C. E. Bygrave, C. to G., Blackburn. June 4.

Matron for Children's Homes (£85), Birmingham Union. Mr. R. J. Curtis, C. to G., Birmingham. May 28.

Night Charge Nurse (£30), Plymouth Guardians. W. H. Davy, C. to G., Greenbank Road, Plymouth. May 28.

Night Sister (£33), Wakefield Union. H. Beaumont, C. to G., 47 Kirkgate, Wakefield. June 4.

